# Functional Connectome before and following Temporal Lobectomy in Mesial Temporal Lobe Epilepsy

**DOI:** 10.1038/srep23153

**Published:** 2016-03-22

**Authors:** Wei Liao, Gong-Jun Ji, Qiang Xu, Wei Wei, Jue Wang, Zhengge Wang, Fang Yang, Kangjian Sun, Qing Jiao, Mark P. Richardson, Yu-Feng Zang, Zhiqiang Zhang, Guangming Lu

**Affiliations:** 1 Center for Information in BioMedicine, Key Laboratory for Neuroinformation of Ministry of Education, School of Life Science and Technology, University of Electronic Science and Technology of China, Chengdu 610054, China; 2 Center for Cognition and Brain Disorders and the Affiliated Hospital, Hangzhou Normal University, Hangzhou 310015, China; 3 Zhejiang Key Laboratory for Research in Assessment of Cognitive Impairments, Hangzhou 310015, China; 4 Laboratory of Cognitive Neuropsychology, Department of Medical Psychology, Anhui Medical University, Hefei 230000, China; 5 Department of Medical Imaging, Jinling Hospital, Nanjing University School of Medicine, Nanjing 210002, China; 6 Department of Medical Imaging, Nanjing Drum Tower Hospital, the Affiliated Hospital of Nanjing University Medical School, Nanjing 210008, China; 7 Department of Neurology, Jinling Hospital, Nanjing University School of Medicine, Nanjing 210002, China; 8 Department of Neurosurgery, Jinling Hospital, Nanjing University School of Medicine, Nanjing 210002, China; 9 Department of Radiology, Taishan Medical University, Tai’an 271016, China; 10 Institute of Psychiatry, Kings College London, London, United Kingdom

## Abstract

As mesial temporal lobe epilepsy (mTLE) has been recognized as a network disorder, a longitudinal connectome investigation may shed new light on the understanding of the underlying pathophysiology related to distinct surgical outcomes. Resting-state functional MRI data was acquired from mTLE patients before (n = 37) and after (n = 24) anterior temporal lobectomy. According to surgical outcome, patients were classified as seizure-free (SF, n = 14) or non-seizure-free (NSF, n = 10). First, we found higher network resilience to targeted attack on topologically central nodes in the SF group compared to the NSF group, preoperatively. Next, a two-way mixed analysis of variance with between-subject factor ‘outcome’ (SF vs. NSF) and within-subject factor ‘treatment’ (pre-operation vs. post-operation) revealed divergent dynamic reorganization in nodal topological characteristics between groups, in the temporoparietal junction and its connection with the ventral prefrontal cortex. We also correlated the network damage score (caused by surgical resection) with postsurgical brain function, and found that the damage score negatively correlated with postoperative global and local parallel information processing. Taken together, dynamic connectomic architecture provides vital information for selecting surgical candidates and for understanding brain recovery mechanisms following epilepsy surgery.

Mesial temporal lobe epilepsy (mTLE) is the most common type of epilepsy in adults^1^. Although epilepsy surgery is a highly effective treatment for drug-resistant mTLE patients, roughly 30% of surgically treated patients experience seizure recurrence^2^,^3^. Potential reasons for seizure relapse after surgery are complex, such as insufficient resection of epileptogenic tissue, and widespread ipsilateral and bilateral temporal lobe functional and structural abnormalities^4^,^5^,^6^.

The risk factors associated with seizure recurrence are unknown, even the underpinning pathology is relatively clear in mTLE^7^. Certain features of the epileptogenic lesion, bilateral atrophy, and remote lesions predict outcome: hippocampal volume asymmetry is a strong predictor of outcome^8^; ipsilateral diffuse hippocampal atrophy and contralateral regional hippocampal atrophy predict poor outcome^9^,^10^. Certain clinical features of the patient predict outcome: older age at surgery, longer epilepsy duration, secondary generalized seizures – all predict poor outcome^11^. But no features predict outcome with strong predictive value, therefore there must be further factors not yet identified.

As widespread structural and functional abnormalities are observed in mTLE^12^,^13^, it has been suggested that mTLE may be better characterized as a network disorder^14^,^15^. A recent anatomical study suggested that large-scale brain network organization may be a predictive factor for outcome^16^. Functional connectivity studies found abnormal topological organization in TLE^17^,^18^, suggesting that brain connectome is an effective way for understanding the functional alteration. Consequentially, assessing the global disturbances of brain function^19^,^20^, rather than evaluating the planned resection area in isolation, may shed new light on estimating probability of seizure freedom after surgery.

To characterize the brain recovery mechanism after surgery, a recent voxel-based morphometric study of seizure-outcome groups^21^ reported significant gray matter recovery in the seizure control group but little in the recurrence group^21^, suggesting that divergent postsurgical alterations in brain structure determine long-term recovery. However, the functional neural network reorganizations leading to seizure freedom or recurrence remain poorly understood^22^.

We aimed to describe the underlying explanation for distinct surgical outcomes using resting-state functional connectivity and graph theoretical analyses. Based on the previous demonstration that pre-surgical brain connections are related to postsurgical outcome^16^, we hypothesized that the successfulness of surgical resection (or attack) could be associated with the pre-surgical brain resilience. The resilience of the brain network, characterized by the degree to tolerance against targeted attacks, is usually associated with the stability of complex brain network^23^. It provides a quantitative insight into relevant network robustness against pathological attacks by disease^24^,^25^. Furthermore, we tracked the dynamic alteration of functional network after surgery, and hypothesized that regions showing divergent reorganization in seizure-free and non-seizure-free groups may provide vital information for understanding distinct surgical outcomes.

## Methods

### Participants

A total of 37 patients with drug-resistant unilateral mTLE participated in this study. Mesial TLE was diagnosed according to International League Against Epilepsy 2001 criteria and based on a comprehensive preoperative evaluation, including seizure history and semiology, neurologic examination, diagnostic MR imaging, and (video) electroencephalography. Detail of these clinical information for each patient was summarized in Table 1. All patients underwent standard anterior temporal lobectomy at Jinling Hospital from June 2009 to December 2013. All patients had hippocampal sclerosis ipsilateral to the seizure focus as revealed by MRI and confirmed by postoperative histopathology. We classified surgical outcome at least 1 year after surgery for all patients according to the Engel category. Patients were separated into two groups: seizure-free (Engel’s: I) with 23 patients (11 left-sided and 12 right-sided) and non-seizure-free (Engel’s: II, III and IV) with 14 patients (7 left-sided and 7 right-sided). Group demographic and clinical information are detailed in Table 2.

Written informed consent was obtained from all participants. The study was approved by the local medical ethics committee at Jinling Hospital, Nanjing University School of Medicine. All the methods were carried out in accordance with the approved guidelines.

### Data acquisition

We acquired functional and structural images using a Siemens Trio 3T scanner (Siemens, Erlangen, Germany) at Jinling Hospital. Resting-state functional images were acquired using a single-shot, gradient-recalled echo planar imaging sequence (255 volumes, repetition time = 2000 ms, echo time = 30 ms, flip angle = 90°, field of view = 240 × 240 mm^2^, inter-slice gap = 0.4 mm, voxel size = 3.75 × 3.75 × 4 mm^3^, 30 transverse slices aligned along the anterior–posterior commissure). We instructed subjects simply to rest with their eyes closed, not to think of anything in particular, and not to fall asleep. Foam padding was used to minimize head motion. Subsequently, we acquired high-resolution 3D T1-weighted anatomical images in the sagittal orientation using a magnetization-prepared rapid gradient-echo sequence (repetition time = 2300 ms, echo time = 2.98 ms, flip angle = 9°, field of view = 256 × 256 mm^2^, voxel size = 0.5 × 0.5 × 1 mm^3^, 176 slices without inter-slice gap). After scanning, subjects were asked whether they had fallen asleep during the scan.

### Lesion mapping

**Figure 1:**
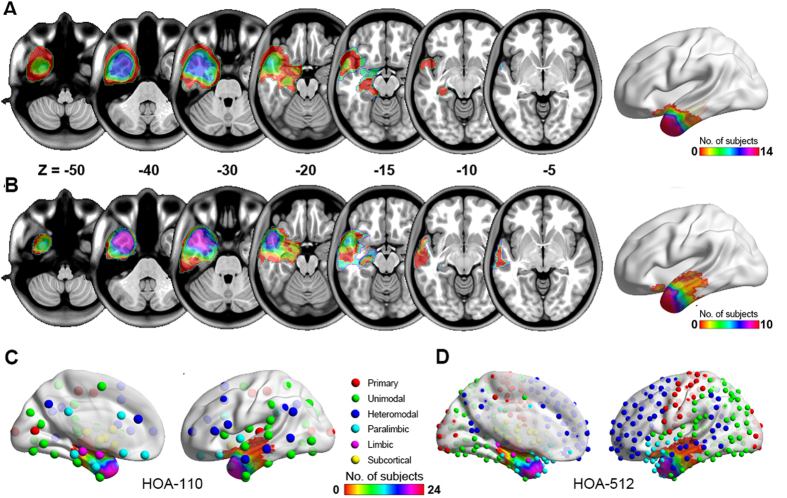
Degree of surgical lacuna overlap across patients. (**A**) Seizure-free patients (n = 14). (**B**) Non-seizure-free patients (n = 10). The surgical lacuna overlap across 24 patients was rendered on the brain surface with HOA-110 (**C**) and HOA-512 (**D**) parcellation schemes, respectively.

Two investigators (J.G. J. and W. W.) independently traced the surgical lacuna manually on postoperative 3D T1-weighted anatomical images, creating an individual volume of interest (VOI) for each patient that was used to quantify the volume of resection. The degree of VOI overlap between investigators was estimated by the Dice coefficient (mean ± SD: 0.89 ± 0.04). The union of all the VOIs were finally used for further analysis (Fig. 1).

### Data preprocessing

Functional image preprocessing was carried out using the DPARSF, REST (www.restfmri.net) and the SPM8 (www.fil.ion.ucl.ac.uk/spm) toolkits. We first flipped (left-to-right flipping) the images of right mTLE patients to allow analysis of both right and left mTLE patients as a homogeneous group, thereby improving statistical power and facilitating investigation of common pathophysiological mechanisms^21^. After exclusion of the first 5 dummy images, functional images were first corrected for temporal differences by slice-timing and for head motion by realignment. No translation or rotation parameters in any given data set exceeded ±1.5 mm or ±1.5°. Moreover, the mean frame-wise displacement (FD) was computed by averaging FD from every time point for each subject^26^. There was no difference in mean FD between the two patient groups (t = 0.32, P = 0.75). Spatial normalization of preoperative functional images was accomplished by 3D T1-based transformation. We coregistered the individual 3D T1 images to functional images. The 3D T1 images were segmented and normalized to Montreal Neurologic Institute (MNI) space by a 12-parameter nonlinear transformation. These transformation parameters were applied to preoperative functional images. For normalization of postoperative images, we additionally used a cost-function modification to exclude the area of the lacuna during the process and avoid bias during the transformations. The implementation of this processing is available in SPM8 and has been adopted in a number of studies on brain images with lesions^21^,^27^. After spatial normalization, pre- and postoperative functional images were resampled at 3 × 3 × 3 mm^3^ voxel size. To avoid introducing artificial correlations, no spatial smoothing was applied as previously suggested^23^,^28^,^29^.

To remove spurious sources of variance, functional images were preprocessed as follows: first, linear trends were removed from time series and temporal band-pass filtering (0.01–0.08 Hz) was performed; second, 24 head motion parameters^30^, averaged signals from cerebrospinal fluid, white matter, and global brain signals were removed by regression^31^,^32^,^33^.

### Reconstruction of functional brain networks

#### Anatomical parcellation

To determine the nodes of the functional network, we used the Harvard-Oxford Atlas (HOA) (112 non-cerebellar anatomical regions of interest [ROIs])^34^. Here we excluded the brainstem, resulting in 55 ROIs for each hemisphere. This coarse parcellation scheme is referred to as HOA-110 (Fig. 1, Table S1). Considering that the range of nodal scales may result in considerable variation of graph theoretical parameters of the functional network^29^,^35^, we also used a high-resolution parcellation network with 512 ROIs of approximately equal size across both hemispheres (http://andrewzalesky.com)^36^. This fine-grained parcellation scheme is referred to as HOA-512. These two parcellation schemes were applied in parallel to the following network analyses for cross-validation of our results.

#### Network construction

We obtained a temporal correlation matrix (of elements r_ij_) for each subject by computing Pearson correlation coefficients between the processed time series of every pair of ROIs. To construct weighted functional connectivity networks, weighted edges (w) were incorporated, representing absolute functional connectivity strength values between connected ROIs, e.g. w_ij_ = |Z_ij_|, where Z_ij_ is the transformed correlation coefficient for node i and j by Fisher’s Z transformation.

#### Network topology analysis

We evaluated the following global network measures: total connection strength (S_net_), overall clustering coefficient (C_net_), global efficiency (E_net_), and small-worldness (Sigma). Nodal topological characteristics were also calculated for each node, including nodal efficiency (E_i_), nodal clustering coefficient (C_i_), and betweenness centrality (BC_i_). The definition and interpretation of these metrics are described in the Supplementary Material.

### Statistical Analyses

**Figure 2:**
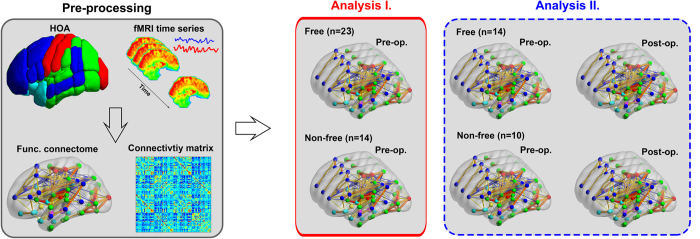
Overview of the analytic strategy. First, cortical and subcortical brain regions were parcellated according to the Harvard-Oxford atlas. Next, a weighted connectivity matrix was constructed from preprocessed functional data. Then, we compared the preoperative functional connectome of seizure-free and non-seizure-free groups (Analysis I); and performed two-way mixed ANOVA for the pre- and postoperative functional connectomes of the two patient groups (Analysis II).

To examine whether functional network architecture is distinct in seizure-free and non-seizure-free groups before surgery, and how functional network architecture reorganized after surgery, we performed two types of analyses including the following steps (Fig. 2). To examine the effect of surgical lacuna on postsurgical brain function, we also correlated the surgical damage score (DS)^37^,^38^ and small-worldness of the postsurgical networks.

#### Network resilience analysis (analysis I)

We evaluated the resilience of the networks by random failure and targeted attack analysis^23^,^39^ for both HOA-110 and HOA-512 schemes. The network cost is defined as the total number of connections divided by the number of all possible connections. We selected a fixed cost (=0.14) that ensured the largest connected component of each functional connectivity network (>95%) with a minimum number of spurious edges^40^.

In random failure analysis, we successively removed randomly chosen nodes until complete network dissolution. This step was repeated 1000 times^23^,^39^. In targeted attack analysis, we first computed the node BC_i_ for all nodes in the network. Then we removed the node with the highest BC_i_. All BC_i_ values were then re-computed and again the node with the highest BC_i_ was removed until one last node remained^23^,^39^. For both random failure and targeted attack analysis, the number of nodes removed per step was set to one for the HOA-110 network and four for the HOA-512 network. At each step after random or targeted node removal, we calculated network measures, including the size of the largest connected component, overall clustering coefficient (C_net_), and global efficiency (E_net_) of the remaining network.

Network resilience was compared between patient groups using two-sample *t*-test. We corrected the statistical significance for multiple comparisons using a false-positive adjustment. Specifically, we set p < (1/n), where n corresponds to the number of network node removal steps as described in our previous study^41^. This implies accepting less than one false-positive per analysis^35^,^42^. Further, we also calculated the area under the curve (AUC) for network resilience, providing an overall resilience estimate independent of the number of steps (node removals) required for network dissolution during random failure or targeted attack analysis.

#### Dynamical functional network reorganization (analysis II)

To examine how functional architecture reorganized according to surgical outcome (seizure-free or non-seizure-free), we performed a two-way mixed analysis of variance (ANOVA) (http://mrtools.mgh.harvard.edu/index.php/GLM_Flex) for network measures. The between-subject ‘random’ factor was ‘outcome’ (seizure-freedom vs. non-seizure-freedom) and the within-subject ‘fixed’ factor was ‘treatment’ (pre-operation vs. post-operation). To compare brain networks between groups, they must have the same number of nodes (ROIs)^43^. For both pre- and postoperative data, we excluded ROIs that overlapped with the surgical lacuna, resulting in 96 non-damaged ROIs for the HOA-110 network, and 474 non-damaged ROIs for the HOA-512 network.

For connectivity-based (edge) statistics, the significant edges in the correlation matrix (present at least in one of the four group datasets) were entered into a two-way mixed ANOVA. The statistical significance for an interaction effect was corrected using a false-positive adjustment^35^,^42^. Specifically, we set p < (1/n), where n is the number of edges.

For global and nodal topological characteristics statistics, we selected a range of cost threshold (0.10 ≤ cos t ≤ 0.35, step = 0.01) for functional connectivity networks in both the HOA-110 and HOA-512 schemes since there is currently no formal consensus regarding selection of thresholds^43^. Further, we calculated the AUC for the global and nodal network metrics, providing an overall estimate for the topological characterization of brain networks independent of cost threshold. Finally, the AUC of global and nodal topological characteristics were entered into a two-way mixed ANOVA. The statistical significance for an interaction effect was corrected using a false-positive adjustment^35^,^42^. Specifically, we set p < (1/n), where n corresponds to the number of nodes.

#### Surgical damage associated with postoperative small-worldness

We correlated the surgical DS and the AUC for small-worldness (0.10 ≤ cos t ≤ 0.35, step = 0.01) of the postoperative networks. The DS was computed as in previous studies^37^,^38^ with some modifications. First, the extent of the surgical lacuna for each individual was quantified by counting the percentage of voxels in each network ROI that overlapped with the lacuna. Second, each percentage was then multiplied by the preoperative nodal topological characteristics (E_i_, and C_i_) of the corresponding ROI, resulting in one value for each damaged ROI. The processes can be expressed as follows:


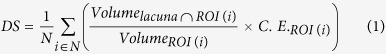


where N is the number of ROIs damaged by surgery and i is one of these ROIs. C.E. represents the preoperative node feature (Ci or Ei) of the given ROI. We calculated two DS values, Ci-based and Ei-based, depending on the nodal feature and correlated C_i_- and E_i_-based DS values with postoperative small-worldness using Pearson’s correlation (P < 0.05).

## Results

### Clinical data

There were no significant differences in preoperative or postoperative values between groups, including mean age, gender proportion, seizure onset age, and duration of epilepsy. Importantly, the volume of the surgical lacuna in the seizure-free group did not differ from that in the non-seizure-free group (t = 0.39, P = 0.70) (Table 2).

### Network resilience before surgery

**Figure 3:**
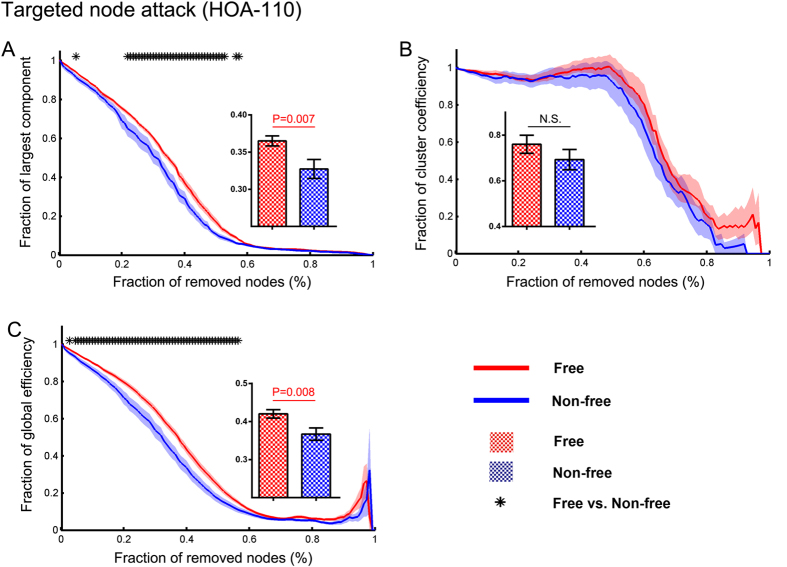
Network resilience analysis. Graphs display the network features as a function of removed nodes. All the features (largest component, clustering coefficient, and global efficiency) were normalized to the measures obtained from the intact network. “Asterisks” illustrate measures that were statistically significant between seizure-free and non-seizure-free groups for each level network attack (P < 0.05 corrected). Shaded bands denote SEM across subjects. Bar graphs represent the resilience of the area under the curve of patient groups.

The two surgical outcome groups showed similar network resilience to random failure but differential resilience to targeted attack. Using the HOA-110 parcellation scheme, we found that the seizure-free group exhibited a higher resilience of the largest component and greater E_net_ across a large range of steps (node removals) compared to the non-seizure-free group (P < 0.05 corrected for multiple comparisons) (Fig. 3). Comparison of the AUC also showed higher resilience of the largest component and greater E_net_ in the seizure-free group. No significant difference was found for the resilience of C_net_. Using the HOA-512 parcellation scheme, all these findings were well reproduced (Fig. S1).

### Network reorganization after surgery

**Figure 4:**
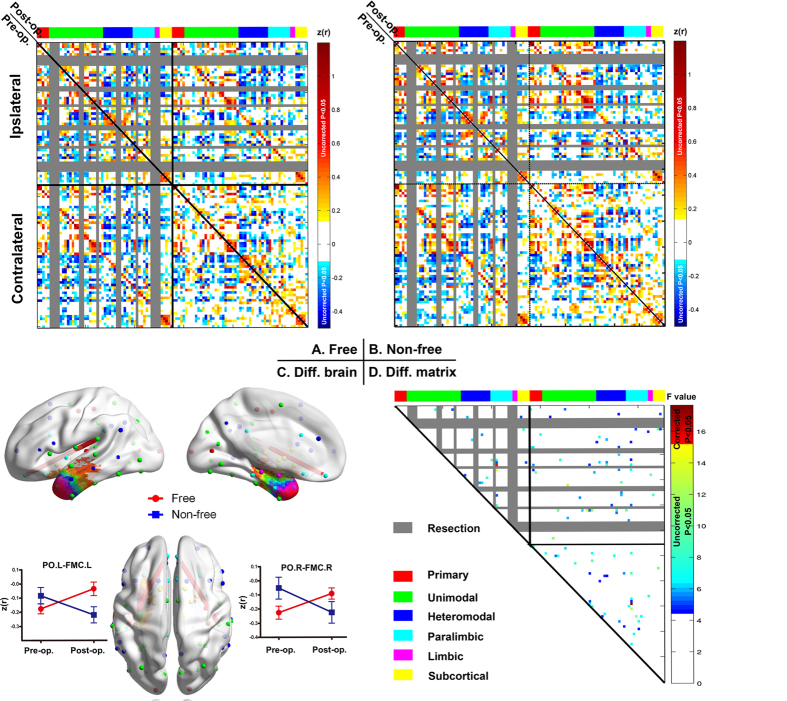
Interaction effect (treatment by outcome) for network edges. (**A,B**) The pre- (lower diagonal) and postoperative (upper diagonal) correlation matrix for seizure-free (**A**) and non-seizure-free groups (**B**). (**C**) Two connections (edges) with significant interaction effects are shown in a 3D brain template (P < 0.05 corrected) by line segment in red. Line graphs show how these connections were altered by surgery in each group. (**D**) Matrix showing connections with significant interaction effect, both corrected (in red) and uncorrected (colored blue to yellow). Columns and rows (nodes) of the matrices are ordered and colored according to six distinct functional modules (Fig. 1, Table S1). The nodes damaged by surgery are shown in gray. PO.L = left parietal operculum cortex, FMC.L= left frontal medial cortex, PO.R = right parietal operculum cortex, SC.R = right subcallosal cortex, PO.L = left parietal operculum cortex.

In analysis II, we investigated how brain functional architecture reorganized in patients achieving seizure freedom compared to those who did not. With the HOA-110 parcellation scheme, we observed a significant ‘outcome’ × ‘treatment’ interaction for edges (Fig. 4). The two patient groups showed divergent alterations after surgery in two edges (all interaction effect, P < 0.05 corrected for multiple comparisons across all edges) connecting left parietal operculum cortex (POC) and left frontal medial cortex (FMC) (F = 15.75, P = 0.0007), right POC and right FMC (F = 17.68, P = 0.0004). Significant ‘outcome’ main effect of connectivity was found between right subcallosal cortex and anterior division of left supramarginal gyrus (with higher strength in seizure-free patients; t = 3.80, P = 4.5 × 10^−4^). ‘Treatment’ main effect indicated connections increased after surgery between: right frontal operculum cortex and left Heschls gyrus (t = 4.09, P = 2.4 × 10^−4^), posterior division of right superior temporal gyrus and left Heschls gyrus (t = 4.48, P = 9.3 × 10^−5^), posterior division of left cingulate gyrus and bilateral cuneal cortex (left, t = 4.21, P = 1.8 × 10^−4^; right, t = 3.99, P = 3.1 × 10^−4^), left supracalcarine cortex and right precuneus cortex (t = 3.95, P = 3.4 × 10^−4^) (Fig. S2).

**Figure 5:**
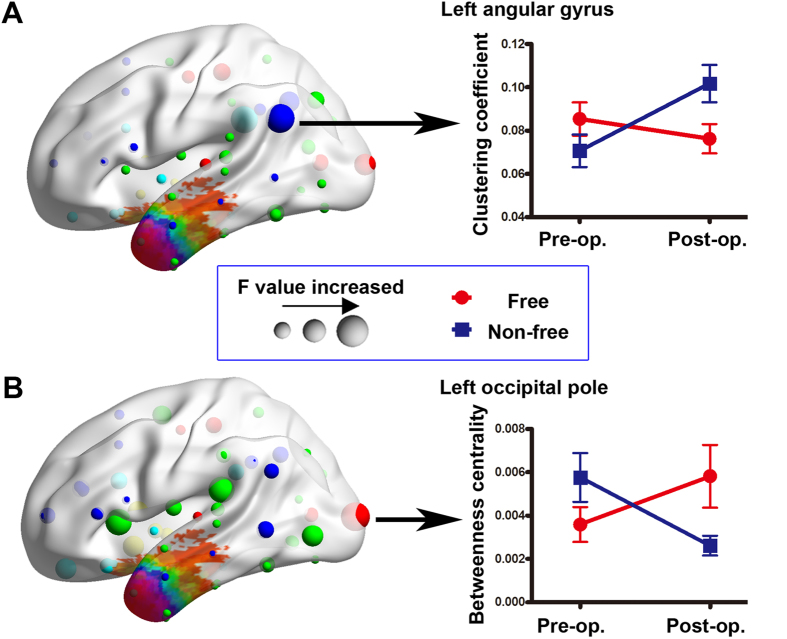
Interaction effect (treatment by outcome) for network nodes. Two nodes show a significant interaction effect. Line graphs show how the two nodes are altered by surgery in each group. The spheres are classified into six modules and colored as in Fig. 1.

The AUC of nodal topological characteristics (efficiency, clustering coefficient, and betweenness centrality) were entered separately into a two-way mixed ANOVA. With the HOA-110 parcellation scheme, we observed significant ‘outcome’ × ‘treatment’ interaction (P < 0.05 corrected for multiple comparisons across all ROIs) of BC_i_ (in left occipital pole [OP] [F = 9.26, P = 0.006]), and C_i_ (in left angular gyrus [F = 8.06, P = 0.01]) (Fig. 5). E_i_ did not show a significant ‘outcome’ × ‘treatment’ interaction. The HOA-512 parcellation scheme yielded similar interaction effect in bilateral temporal nodes (Fig. S3). No significant group main effect was found. Significant time main effect was found in left precuneus cortex (with decreased BC_i_ after surgery; t = −3.04, P = 0.003) (Fig. S4).

For global topological characteristics, only S_net_ showed significant interaction effect in both the HOA-110 (F = 4.84, P = 0.04) and the HOA-512 (F = 4.12, P = 0.05) parcellation scheme. Post-hoc analysis indicated NSF patients showed higher S_net_ than SF patients (t = 2.65, P = 0.01 for HOA-110; t = 2.47, P = 0.02 for HOA-512). No significant interaction and main effect was observed for all the other global characteristics.

### Surgical damage and postoperative network properties

**Figure 6:**
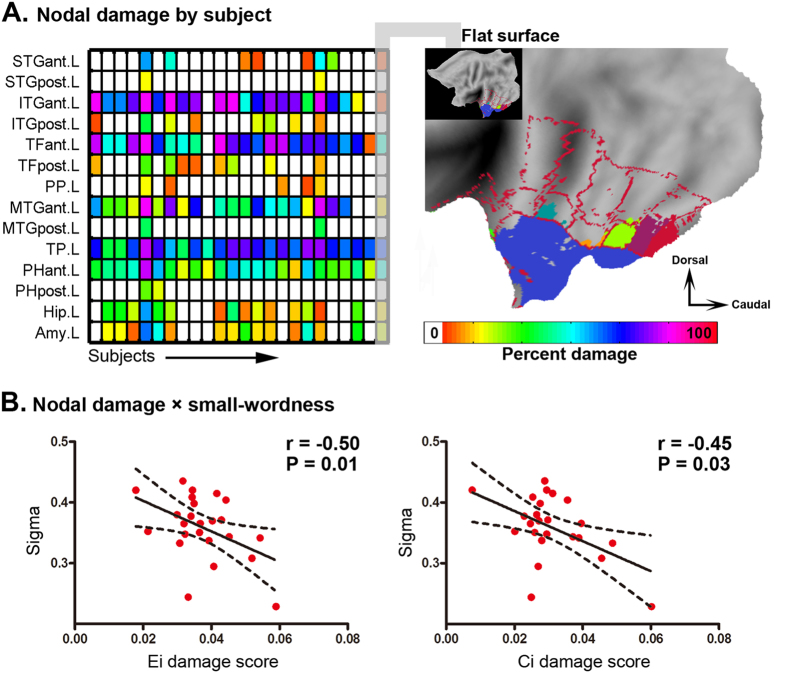
Clinical damage and postsurgical small-worldness. (**A**) The matrix shows the percentage of each node damaged in each subject. To illustrate how this was percentage computed, the resected volumes of one subject are overlapped on a flat surface with red circles showing the border of the damaged ROIs. (**B**) Both E_i_- and C_i_-based damage scores are significantly and negatively correlated with the postsurgical small-worldness (sigma). STGant. = anterior part of superior temporal gyrus, STGpost. = posterior part of superior temporal gyrus, ITGant. = anterior part of inferior temporal gyrus, ITGpost. = posterior part of inferior temporal gyrus, TFant. = anterior part of temporal fusiform cortex, TFpost. = posterior part of temporal fusiform cortex, PP. = planum polare, MTGant. = anterior part of middle temporal gyrus, TP. = temporal pole, PHant. = anterior part of parahippcampal gyrus, PHpost. Posterior part of parahippcampal gyrus, Hip. = hippocampus, Amy. = amygdala, L = left hemisphere. E_i_ and C_i_ represent the global efficiency and clustering coefficient respectively.

There was no significant group difference in E_i_-based damage score (t = 0.17, P = 0.87), consistent with the approximately equal mean lacuna volumes. In contrast, C_i_-based damage score (t = 2.17, P = 0.04) was higher in NSF patients. In addition, we found that the small-worldness of the postoperative brain functional network negatively correlated with both E_i_-based (r = −0.51, P = 0.01) and C_i_-based (r = −0.45, P = 0.03) damage score (Fig. 6).

## Discussion

A core characteristic of epilepsy is the excessively spontaneous neuronal activity. Resting-state fMRI, particularly focusing on the spontaneous brain activity, has particular advantage to illustrate functional alteration in epileptic brain. Using resting-state fMRI, a number of studies discussed the epileptic network, trying to characterize the pathophysiology mechanism^44^,^45^,^46^,^47^,^48^,^49^, uncover the neuronal mechanism of cognitive deficit^50^, or discriminate epilepsy patients from heathy subjects^51^. In the current study, we focused on the brain functional feature in patients with distinct surgical outcomes. Using resting-state fMRI and graph-based network analysis, we revealed the functional connectomic features in mTLE patients with distinct surgical outcomes. Preoperatively, the seizure-free group showed higher network resilience than the non-seizure-free group, underscoring the potential predictive value of this global network measurement. Furthermore, the two surgical outcome groups exhibited divergent postsurgical reorganization of nodal topological characteristics, mainly in the temporoparietal junction (TPJ), as well in its connection with ventral prefrontal cortex, suggesting that changes in extratemporal circuits associating with recovery and relapse. Finally, postoperative network property was negatively associated with surgical damage. These findings extend our understanding of the pathophysiological mechanisms underlying the distinct outcomes of mTLE surgery.

### Presurgical resilience to network perturbation

A comprehensive presurgical assessment of mTLE patients is crucial to weigh the possible benefits (e.g., seizure control) against the risks (e.g., postsurgical complications or cognitive deficits)^52^. Many neuroimaging studies have sought to predict the extent of seizure control and cognitive damage after surgery^21^,^39^,^53^,^54^,^55^,^56^. Bonilha and colleagues reported a relationship between the small-worldness of the presurgical structural network in mTLE patients and surgical outcome^16^. In the current study, we found that the largest component and global efficiency of seizure-free patients showed higher resilience to targeted attack than non-seizure-free patients, regardless of the parcellation scheme (HOA-110 or HOA-512). Targeted attack measures the brain resilience to simulated sequential elimination of topologically central nodes^23^, suggesting that pathological attack on the network hub will have a disproportionately larger impact on network information processing^57^. Targeted attack analysis has been applied to a number of clinical disorders, such as Alzheimer’s disease^24^ and autism^25^. Previous network studies found that mTLE patients showed abnormal resilience to targeted attack for both structural^39^ and functional networks^58^. We found a significant association between brain network resilience and surgical outcome, suggesting that there are more or stronger connections in seizure-free patients^25^, allowing enduring network integrity even after attack. These abundant connections could be a long-term result of recurrent seizures. In contrast, clustering coefficient resilience was not significantly different between patient groups, suggesting that the extent of local network cliquishness or efficiency of information transfer^59^ is as robust in non-seizure-free patients as in seizure-free patients, and further that the predictive value of clustering coefficient resilience is limited.

### Dynamic alterations of regional/connectivity characteristics

To elucidate brain functional network reorganization patterns leading to seizure freedom or recurrence, we compared the dynamic alterations of seizure-free patients to those of non-seizure-free patients. The results are in accordance with previous studies demonstrating that the ventral PFC is a vital part of the epileptogenic network^60^,^61^,^62^. Using depth electrodes to record seizure propagation in mTLE patients, Lieb *et al*.^60^ observed a common pattern, with the seizure initially invading the ipsilateral ventral PFC and then spreading to the contralateral side^60^. A neuroimaging study adopting diffusion tensor imaging tractography found decreased structural connectivity between ipsilateral frontal and temporal cortices in mTLE patients^61^. In line with this result, we found that increased functional connectivity between ventral medial PFC and TPJ following surgery was associated with seizure freedom. A PET study found that the metabolic activities of the ventral PFC and temporal lobe were abnormal in mTLE patients^62^. In addition, we found a divergent alteration in ipsilateral TPJ (the C_i_ of the angular gyrus) between the two patient groups. Using the finer HOA-512 parcellation scheme, we confirmed the findings in the TPJ area. Both ventral medial PFC and TPJ are belong to the default mode network (DMN). The abnormal function of DMN in mTLE patients has been reported in local activity^47^, within-network connectivity^63^ and causal connectivity with mesial temporal lobe^44^. Our findings further suggested the postoperative functional alteration of DMN may be an important biomarker for surgical outcome. Although anterior temporal lobectomy can ameliorate drug-resistant mTLE, 60–100% of patients will suffer a postoperative visual field defect^64^. Left occipital pole, a central pivot of the visual system, showed divergent alterations, implicating the disruption of visual system may be different between SF and NSF patients.

### Clinical damage and postsurgical small-worldness

To examine whether postoperative brain function was related to surgical damage, we computed a damage score for each patient and analyzed the correlation with the postoperative network small-worldness (sigma) value across patients. As in the study of Gratton *et al*.^37^, this damage score not only reflected the size of the surgical lacuna but also the function importance of the damaged cortical regions. In our study, postoperative small-worldness decreased as the damage score increased, implying that surgeons may be able to predict postsurgical function if they can precisely outline the resection area before surgery. The small-worldness of human brain networks has been related to a number of cognitive processes, such as intelligence^65^, memory^66^ and attention^67^. Future studies should examine the association of the damage score with more specific cognitive functions using behavioral measures.

### Methodological considerations

Large-scale brain connectome modeling and analysis is a rapidly developing research field, but there are still controversies concerning optimal analytic strategies^68^. Thus, the present brain functional network analysis employed several parallel schemes. First, two resolution parcellation schemes were used to cross-validate our findings. However, analysis still produced some inconsistent results. Such divergence may result from the different node scales^36^,^69^. Second, in this study we used weighted networks, reflecting heterogeneity in capacity and strength of connectivity, but binary networks are simpler to use for statistical comparisons^70^. Finally, we used multiple cost thresholds to evaluate the stability of the topological organization in brain functional networks because there is currently no formal consensus regarding cost threshold selection.

### Limitations

The present results should be interpreted in the context of the study limitations. First, due to the small sample size, we grouped left and right mTLE patients into one group, although previous studies suggested that the side of seizure onset influences the abnormal connectivity features of mTLE^58^,^71^. Thus, our findings should be understood as revealing pathophysiology changes common to left and right mTLE rather than mechanisms specific for left or right mTLE patients. Second, the interval between operation and follow-up MRI examination was relatively short. As the outcome may change even after 10 years^72^, a longer-term longitudinal study is necessary. Third, although we balanced the major demographic and clinical factors between groups (age, gender proportion, volume of surgical lacuna, and follow-up interval), preoperative cognitive functions, such as memory and language ability, were not examined^53^,^73^. Finally, all the findings were derived from group-level statistical results. To what extent these findings can be applied for individual preoperative estimates requires further investigation.

## Conclusions

In current study, we combined resting-state functional connectivity and graph theoretical approaches to investigate the pathophysiology of mTLE patients with different surgical outcomes. Relatively high preoperative network resilience could provide complementary information to screen for patients most likely to achieve seizure freedom after surgery. Re-wiring of the ventral prefrontal cortex and temporoparietal junction area appears to be the predominant postsurgical functional reorganization mechanism influencing outcome. In summary, these findings may help to establish better surgical referral criteria and contribute to our understanding of brain recovery following surgery for mTLE.

## Additional Information

**How to cite this article**: Liao, W. *et al*. Functional Connectome before and following Temporal Lobectomy in Mesial Temporal Lobe Epilepsy. *Sci. Rep*. **6**, 23153; doi: 10.1038/srep23153 (2016).

## Supplementary Material

Supplementary Information

## Figures and Tables

**Table 1:** Clinical information of each patient.

Pt #	Age/Sex	Anamnesis	StructuralMRI	Seizure type orsemiology	Lateralization by (video) EEG	Surgicaloutcome
1	10/M	Febrile convulsion at 5 years old; intracranial infection at 3 years old	LHS	CPS-SGTCS	L (Front., Ant. Temp.)	I
2	37/M	Febrile convulsion at 4 years old; epidemic encephalitis B	RHS	SPS-CPS	R (Cent., Temp., Front.)	I
3	29/F	No	RHS	SPS-CPS	R (Front., Ant. Temp.)	I
4	26/F	No	RHS	CPS	R (Cent., Par., Post. Temp.)	I
5	19/F	Febrile convulsion at 1 years old	LHS	SPS-CPS	L (Post. Temp.)	I
6	37/M	Febrile convulsion at 1 years old	LHS	SPS-SGTCS	L (Front., Cent., Temp.)	I
7	23/M	No	LHS	CPS-SGTCS	L (Front., Temp.)	I
8	18/M	Febrile convulsion at 1 years old; epidemic encephalitis B	LHS	SPS-CPS-SGTCS	L (Cent., Temp., Front.)	I
9	25/F	Febrile convulsion at 3 years old	RHS	SPS-CPS	R (Temp., Front.)	I
10	17/M	No	RHS	SPS-CPS-SGTCS	R (Cent., Temp., Front.)	I
11	22/F	History of intrauterine hypoxia	RHS	CPS	R (Ant. Temp., Mid. Temp.)	I
12	30/M	No	RHS	CPS-SGTCS	R (Temp., Cent., Front.)	I
13	18/M	Febrile convulsion at 3 years old	RHS	SPS-CPS-SGTCS	R (Temp., Occip.)	I
14	18/M	No	RHS	CPS	R (Front., Temp.)	I
15	15/M	History of head injury at 5 years old	RHS	SPS-CPS	R (Cent. Front.)	I
16	20/F	No	LHS	SPS-CPS	L (Front., Cent., Ant. Temp.)	I
17	17/M	No	LHS	CPS-SGTCS	R (Front., Temp., Par.)	I
18	28/F	Febrile convulsion before 3 years old	RHS	CPS-SGTCS	R (Temp., Front.)	I
19	20/F	Febrile convulsion	LHS	SPS-CPS-SGTCS	L (Temp., Cent., Front.)	I
20	26/M	Febrile convulsion at 5 years old	RHS	CPS-SGTCS	R (Front., Mid. Temp.)	I
21	33/M	No	LHS	SPS-CPS	L (Front., Cent., Ant. Temp.)	I
22	33/M	Febrile convulsion before 3 years old	LHS	SPS-CPS	L (Temp., Front.)	I
23	44/M	No	LHS	GTCS	L (Front., Temp.)	I
24	19/M	No	RHS	CPS-SGTCS	R (Temp., Cent., Front.)	II
25	15/M	Febrile convulsion before 1 years old	RHS	CPS-SGTCS	R (Temp., Front.)	II
26	37/F	epidemic encephalitis B	RHS	SPS-SGTCS	R (Front., Cent., Ant. Temp.)	II
27	22/M	No	LHS	CPS	L (Front., Temp.)	II
28	17/F	Febrile convulsion before 4 years old	LHS	CPS	L (Par., Occip., Post. Temp.)	III
29	26/M	History of encephalitis	LHS	SPS-CPS	L (Front., Cent., Ant. Temp.)	II
30	23/F	No	LHS	SPS-CPS-SGTCS	L (Cent., Temp., Front.)	III
31	49/M	No	RHS	SPS-CPS	R (Ant. Temp.)	III
32	42/F	No	LHS	CPS-SGTCS	L (Cent., Temp.)	II
33	20/F	No	LHS	SPS	L (Ant. Temp.)	III
34	28/F	No	LHS	SPS-CPS-SGTCS	L (Ant. Temp., Front.)	III
35	25/M	Febrile convulsion before 7 years old	RHS	SPS-CPS-SGTCS	R (Front., Cent., Ant. Temp.)	III
36	25/F	Febrile convulsion before 5 years old	RHS	SPS	R (Temp., Front.)	III
37	25/F	Febrile convulsion before 2 years old	RHS	CPS-SGTCS	R (Cent., Par., Temp.)	III

Ant. = anterior; Cent. = central area; CPS = complex partial seizures; Front. = frontal area; L = left hemisphere; Mid. = middle; Occip. = occipital area; Par. = parietal area; Post. = posterior; R = right hemisphere; SPS = simple partial seizures; SGTCS = secondary generalized tonic-clonic seizures; Temp. = temporal area.

**Table 2:** Demographic and clinical characteristics of all patients.

Characteristic	Seizure-free	Non-seizure-free	P value
Preoperative data			
Group size (n)	23	14	—
Age (years)	24.52 ± 8.32	26.64 ± 9.68	0.48^a^
Sex (male/female)	15/8	6/8	0.31^b^
Laterality (left/right)	11/12	7/7	1.00^b^
Duration (years)	13.48 ± 1.3	10.11 ± 5.70	0.11^a^
Onset age (years)	11.09 ± 1.76	16.43 ± 7.57	0.061^a^
Postoperative data			
Group size (n)	14	10	—
Age (years)	23.50 ± 7.76	27.00 ± 11.59	0.38^a^
Sex (male/female)	9/5	5/5	0.48^b^
Laterality (left/right)	6/8	6/4	0.41^b^
Duration (years)	14.07 ± 6.72	11.23 ± 5.76	0.29^a^
Onset age (years)	12.29 ± 1.49	15.70 ± 8.25	0.24^a^
Interval from surgery to postoperative fMRI (months)	13.77 ± 9.04	22.02 ± 16.09	0.12^c^
Surgical lacuna (cm^3^)	5.14 ± 1.66	4.84 ± 1.99	0.70^a^

Values are mean ± SD.

^a^two-sample *t*-test.

^b^χ2 test.

^c^Mann Whitney U-test.
