# Supplementary material for: Functional Connectome before and following Temporal Lobectomy in Mesial Temporal Lobe Epilepsy
Source: Sci Rep. 2016 Mar 22;6:23153. doi: 10.1038/srep23153 (PMC4802388; doi:10.1038/srep23153)
Supplement: Supplementary Information [file srep23153-s1.pdf]

Supplementary Methods for

Functional Connectome before and following Temporal Lobectomy in Mesial Temporal Lobe Epilepsy

Wei Liao[1,2,3†], Gong-Jun Ji[4,2,3†], Qiang Xu[5], Wei Wei[5], Jue Wang[2,3], Zhengge Wang[6], Fang Yang[7], Kangjian Sun[8], Qing Jiao[9], Mark P. Richardson[10], Yu-Feng Zang[2,3], Zhiqiang Zhang [5]*, Guangming Lu[5]*

1. Center for Information in BioMedicine, Key Laboratory for Neuroinformation of Ministry of Education, School of Life Science and Technology, University of Electronic Science and Technology of China, Chengdu 610054, China.
2. Center for Cognition and Brain Disorders and the Affiliated Hospital, Hangzhou Normal University, Hangzhou 310015, China;
3. Zhejiang Key Laboratory for Research in Assessment of Cognitive Impairments, Hangzhou 310015, China;
4. Laboratory of Cognitive Neuropsychology, Department of Medical Psychology, Anhui Medical University, Hefei 230000, China;
5. Department of Medical Imaging, Jinling Hospital, Nanjing University School of Medicine, Nanjing 210002, China;
6. Department of Medical Imaging, Nanjing Drum Tower Hospital, the Affiliated Hospital of Nanjing University Medical School, Nanjing 210008, China;
7. Department of Neurology, Jinling Hospital, Nanjing University School of Medicine, Nanjing 210002, China;
8. Department of Neurosurgery, Jinling Hospital, Nanjing University School of Medicine, Nanjing 210002, China;
9. Department of Radiology, Taishan Medical University, Tai'an 271016, China;
10. Institute of Psychiatry, Kings College London, London, United Kingdom;

†These authors contributed equally to this work.

*Corresponding author: Zhiqiang Zhang, and Guangming Lu, the Department of Medical Imaging, Nanjing Jinling Hospital, 305#, Eastern Zhongshan Rd., Nanjing 210002, PR China. Fax: +86-25-84804659. E-mail: zhangzq2001@126.com (Z. Zhang) or cjr.luguangming@vip.163.com (G. Lu).

Methods
Table S1. Regions of interest (ROI) in the Harvard-Oxford Atlas
Figure S1. Network resilience analysis with HOA-512.
Figure S2. Outcome and treatment main effect of edges.
Figure S3. Interaction effect between outcome and treatment for network nodes (HOA-512).
Figure S4. Treatment main effect of nodes.
References.

Methods

Network topology analysis

We measured the brain functional network topologies using the Brain Connectivity Toolbox (http://www.brain-connectivity-toolbox.net) [1]. We evaluated the following global network measures: 1) total connection strength (S_{net}), 2) overall clustering coefficient (C_{net}), 3) global efficiency (E_{net}), and 5) small-worldness (Sigma). Nodal topological characteristics were also calculated for each node, including nodal efficiency, nodal clustering coefficient, and betweenness centrality. The definition and brief interpretation of these metrics is described below.

Global properties

The degree (S_i^w) was computed as the sum of the weights of all the connections of node i, that is $S_i^w = \sum_{j \in N} w_{ij}$. The degree S_i^w quantifies the extent to which a node is relevant to the graph [1]. The total connection strength S_{net}^w of the network was computed as the sum of S_i^w for all nodes N in the network:

$$S_{net}^w = \frac{1}{N} \sum_{i \in N} S_i^w \ .$$

The nodal efficiency of a given node i (E_i^w) is defined as the inverse of the mean harmonic shortest path length between this node and all other nodes in the network [2, 3], according to the formula:

$$E_i^w = \frac{1}{N-1} \sum_{i \neq j \in N} \frac{1}{L_{ij}}$$

where the L_{ij} is the weighted shortest path length between nodes i and j in the network. E_i^w quantifies the importance of the nodes for the communication within the network [4]. Accordingly, the node i is more important if the value of E_i^w is higher.

The weighted clustering coefficient of node i, C_i^w, which expresses the likelihood that the neighbourhoods of node i are connected [5], is defined as follows:

$C_i^w = \frac{\sum_{j,h \in N} (w_{ij} w_{ih} w_{jh})^{1/3}}{k_i(k_i - 1)}$, where w_{ij} is the weight between nodes i and j in the network, and k_i is the degree of node i. The clustering coefficient is zero, $C_i^w = 0$, if the nodes are isolated or with just one connection. The overall clustering coefficient, C_{net}^w, was computed as the average of C_i^w across all nodes in the network: $C_{net}^w = \frac{1}{N} \sum_{i \in N} C_i^w$, extent measure of the local interconnectivity or cliquishness of the network [6].

The path length between nodes i and j was defined as the sum of the edge lengths along the path, where each edge's length was obtained by computing the reciprocal of the edge weight, $1/w_{ij}$. The shortest path length L_{ij} between nodes i and j was defined as the length of the path with the shortest length between the two nodes. The weight characteristic shortest path length L_{net}^w of a network was measured by a "harmonic mean" length between pairs [7], to overcome the problem of possibly disconnected network components. Formally, L_{net}^w is the reciprocal of the average of the reciprocals:

$$L_{net}^w = \frac{1}{\frac{1}{N(N-1)} \sum_{i=1}^{N} \sum_{j \neq i}^{N} \frac{1}{L_{ij}}},$$

where N is the number of nodes. The weight characteristic shortest path length quantifies the ability for information propagation in parallel.

Small-world properties were originally proposed by Watts and Strogatz [6]. Here, we investigated small-world properties of the weighted functional connectivity network. A small-world network has similar path length but higher clustering than a random network, that is $\gamma = C_{net}^w / C_{random}^w > 1$, $\lambda = L_{net}^w / L_{random}^w \approx 1$ [6]. These two conditions can also be summarized into a scalar quantitative measurement, the small-worldness, $\sigma = \gamma / \lambda$, that is typically larger than one in case of small-world organization [8, 9]. For each individual brain network a set of 100 comparable random networks with similar

degree sequence and symmetric adjacency matrix were formed, and C^{w}_{random} and L^{w}_{random} were defined as the average weighted clustering coefficient and weighted path length.

Nodal characteristics analysis

Three nodal topological characteristics, including clustering coefficient (C_i^w) (see above definition), efficiency (E_i^w) (see above definition) and betweenness centrality (BC_i^w) were used. These measures are known to be interrelated, each provides a different viewpoint from which to discern major features of the large-scale architecture [10, 11].

The betweenness centrality B_i^w of a node i considers the fraction of all shortest paths in the network that pass through the node [12]. In this study, we computed the normalized betweenness as $BC_i^w = B_i^w / \langle B_i^w \rangle$, where $\langle B_i^w \rangle$ is the averaged nodal betweenness of the network. The global centrality measure BC_i^w captures the influence of a node over information flow between other nodes in the network.

Nodes with high clustering coefficient, C_i^w, indicates how close a given node's neighbors are to forming a clique; those with high efficiency, E_i^w, are relevant for information flow; those with high betweenness centrality, BC_i^w, may serve as way stations for network traffic. Accordingly, nodes with these properties were considered as network hubs.

References:

1. Rubinov, M. & Sporns, O. Complex network measures of brain connectivity: Uses and interpretations. *Neuroimage* **52**, 1059-1069 (2010).
2. Achard, S. & Bullmore, E. Efficiency and cost of economical brain functional networks. *PLoS Comput Biol* **3**, e17 (2007).
3. Shu, N.*, et al.* Diffusion tensor tractography reveals disrupted topological efficiency in white matter structural networks in multiple sclerosis. *Cereb Cortex*, doi:10.1093/cercor/bhr1039 (2011).
4. Bassett, D.S. & Bullmore, E. Small-world brain networks. *The Neuroscientist : a review journal bringing neurobiology, neurology and psychiatry* **12**, 512-523 (2006).
5. Onnela, J.P., Saramaki, J., Kertesz, J. & Kaski, K. Intensity and coherence of motifs in weighted complex networks. *Phys Rev E Stat Nonlin Soft Matter Phys* **71**, 065103 (2005).
6. Watts, D.J. & Strogatz, S.H. Collective dynamics of 'small-world' networks. *Nature* **393**, 440-442 (1998).
7. Newman, M.E.J. The structure and function of complex networks. *SIAM review* **45**, 167-256 (2003).
8. Achard, S., Salvador, R., Whitcher, B., Suckling, J. & Bullmore, E. A resilient, low-frequency, small-world human brain functional network with highly connected association cortical hubs. *J Neurosci* **26**, 63-72 (2006).
9. Humphries, M.D., Gurney, K. & Prescott, T.J. The brainstem reticular formation is a small-world, not scale-free, network. *Proc Biol Sci* **273**, 503-511 (2006).
10. Hagmann, P.*, et al.* Mapping the structural core of human cerebral cortex. *PLoS Biol* **6**, e159 (2008).
11. Tian, L., Wang, J., Yan, C. & He, Y. Hemisphere- and gender-related differences in small-world brain networks: a resting-state functional MRI study. *Neuroimage* **54**, 191-202 (2011).
12. Freeman, L.C. A set of measures of centrality based upon betweenness. *Sociometry* **40**, 35-41 (1977).

Table S1. Regions of interest (ROI) in the Harvard-Oxford Atlas

**Region name**	**Abbreviation**	Classification
Precentral Gyrus	PRG.L	Primary
Postcentral Gyrus	POG.L	Primary
Intracalcarine Cortex	CALC.L	Primary
Heschls Gyrus (includes H1 and H2)	HG.L	Primary
Occipital Pole	OP.L	Primary
Superior Temporal Gyrus, anterior division	STGant.L	Unimodal
Superior Temporal Gyrus, posterior division	STGpost.L	Unimodal
Inferior Temporal Gyrus, anterior division	ITGant.L	Unimodal
Inferior Temporal Gyrus, posterior division	ITGpost.L	Unimodal
Inferior Temporal Gyrus, temporooccipital part	ITGto.L	Unimodal
Superior Parietal Lobule	SPL.L	Unimodal
Supramarginal Gyrus, anterior division	SGant.L	Unimodal
Lateral Occipital Cortex, superior division	OLs.L	Unimodal
Lateral Occipital Cortex, inferior division	OLi.L	Unimodal
Supplementary Motor Cortex	SMC.L	Unimodal
Cuneal Cortex	CN.L	Unimodal
Lingual Gyrus	LG.L	Unimodal
Temporal Fusiform Cortex, anterior division	TFant.L	Unimodal
Temporal Fusiform Cortex, posterior division	TFpost.L	Unimodal
Temporal Occipital Fusiform Cortex	TOF.L	Unimodal
Occipital Fusiform Gyrus	OF.L	Unimodal
Frontal Operculum Cortex	FO.L	Unimodal
Central Opercular Cortex	CO.L	Unimodal
Parietal Operculum Cortex	PO.L	Unimodal
Planum Polare	PP.L	Unimodal
Planum Temporale	PT.L	Unimodal
Supracalcarine Cortex	SCLC.L	Unimodal
Frontal Pole	FP.L	Heteromodal
Superior Frontal Gyrus	SFG.L	Heteromodal
Middle Frontal Gyrus	MFG.L	Heteromodal
Inferior Frontal Gyrus, pars triangularis	IFG3t.L	Heteromodal
Inferior Frontal Gyrus, pars opercularis	IFG3o.L	Heteromodal
Middle Temporal Gyrus, anterior division	MTGant.L	Heteromodal
Middle Temporal Gyrus, posterior division	MTGpost.L	Heteromodal
Middle Temporal Gyrus, temporooccipital part	MTGto.L	Heteromodal
Supramarginal Gyrus, posterior division	SGpost.L	Heteromodal
Angular Gyrus	AG.L	Heteromodal
Paracingulate Gyrus	PAC.L	Heteromodal
Precuneus Cortex	PCN.L	Heteromodal
Insular Cortex	INS.L	Paralimbic
Temporal Pole	TP.L	Paralimbic
Frontal Medial Cortex	FMC.L	Paralimbic

Subcallosal Cortex	SC.L	Paralimbic
Cingulate Gyrus, anterior division	CGant.L	Paralimbic
Cingulate Gyrus, posterior division	CGpost.L	Paralimbic
Frontal Orbital Cortex	FOC.L	Paralimbic
Parahippocampal Gyrus, anterior division	PHant.L	Paralimbic
Parahippocampal Gyrus, posterior division	PHpost.L	Paralimbic
Hippocampus	Hip.L	Limbic
Amygdala	Amy.L	Limbic
Thalamus	Tha.L	Subcortical
Caudate	Caud.L	Subcortical
Putamen	Put.L	Subcortical
Pallidum	Pall.L	Subcortical
Accumbens	Accbns.L	Subcortical
Precentral Gyrus	PRG.R	Subcortical

Figure S1

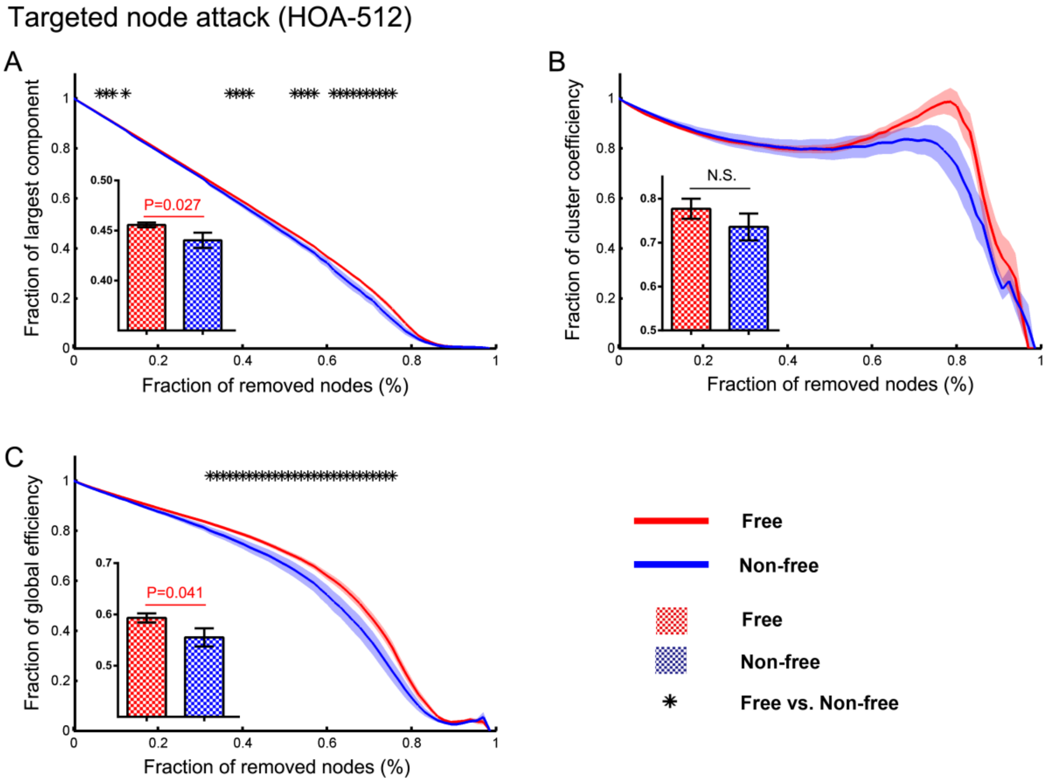

Figure S1. Network resilience analysis with HOA-512. Graphs display the network features as a fraction of removed nodes. All the features (largest component, cluster coefficient and global efficiency) were normalized to the measure obtained from the intact network. "Stars" illustrate measures that were statistically significant between seizure-free and non-seizure-free groups for each level of percent of the network being attacked (P<0.05 corrected). Shadow bands with different colors show the SEM across subjects of the corresponding group. Bar graphs represent the resilience of the area under the curve of patient groups.

Figure S2

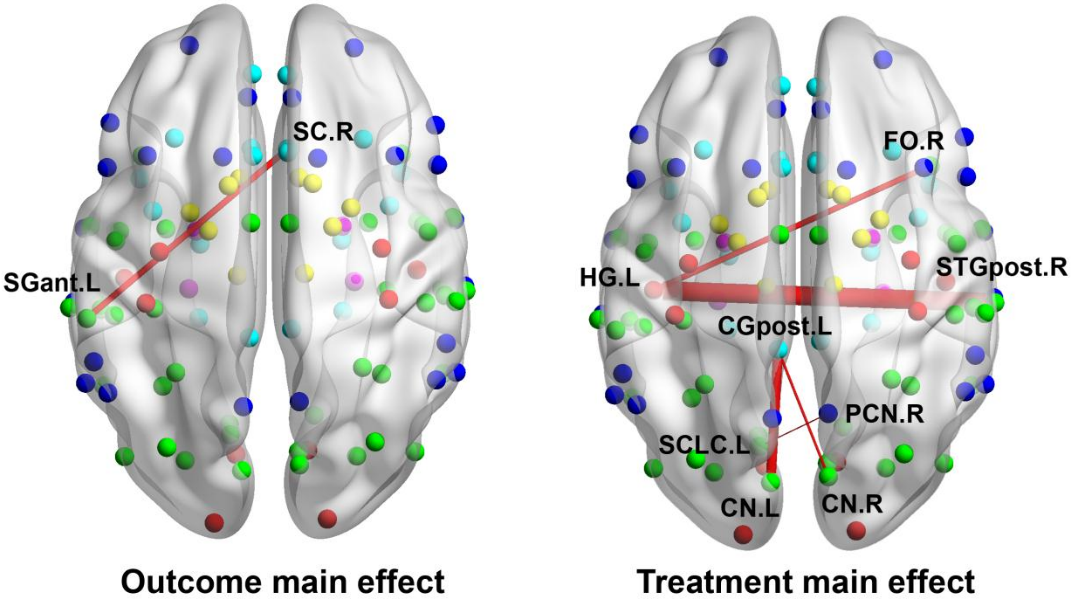

Figure S2. Outcome and treatment main effect of edges. Edges in red color indicate higher strength in seizure-free patients or pre-operative state. The full name of the regions connected by these edges could be found in Table S1.

Figure S3

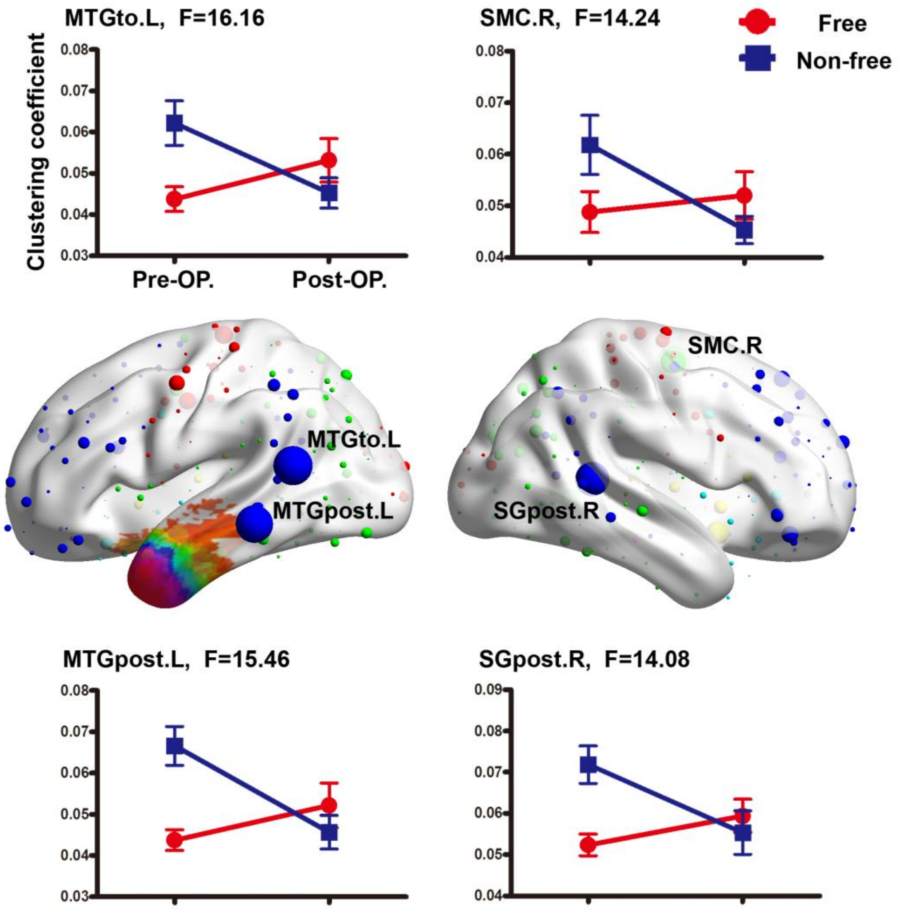

Figure S3. Interaction effect between outcome and treatment for network nodes (HOA-512). Four nodes show significant interaction effect. Line graphs show how the four nodes modulated by surgery in each patient group. The full name of the nodes could be found in Table S1. The spheres are classified into six modules and colored as

Figure S4

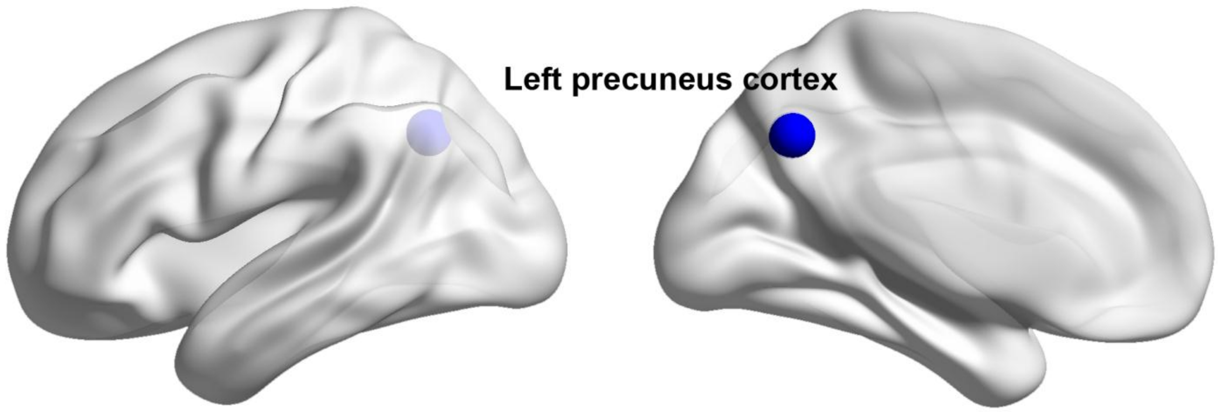

Figure S4. Treatment main effect for betweeness centrality. The full name of the regions connected by these edges could be found in Table S1.

References:

1. Rubinov, M. & Sporns, O. Complex network measures of brain connectivity: Uses and interpretations. *Neuroimage* **52**, 1059-1069 (2010).
2. Achard, S. & Bullmore, E. Efficiency and cost of economical brain functional networks. *PLoS Comput Biol* **3**, e17 (2007).
3. Shu, N.*, et al.* Diffusion tensor tractography reveals disrupted topological efficiency in white matter structural networks in multiple sclerosis. *Cereb Cortex*, doi:10.1093/cercor/bhr1039 (2011).
4. Bassett, D.S. & Bullmore, E. Small-world brain networks. *The Neuroscientist : a review journal bringing neurobiology, neurology and psychiatry* **12**, 512-523 (2006).
5. Onnela, J.P., Saramaki, J., Kertesz, J. & Kaski, K. Intensity and coherence of motifs in weighted complex networks. *Phys Rev E Stat Nonlin Soft Matter Phys* **71**, 065103 (2005).
6. Watts, D.J. & Strogatz, S.H. Collective dynamics of 'small-world' networks. *Nature* **393**, 440-442 (1998).
7. Newman, M.E.J. The structure and function of complex networks. *SIAM review* **45**, 167-256 (2003).
8. Achard, S., Salvador, R., Whitcher, B., Suckling, J. & Bullmore, E. A resilient, low-frequency, small-world human brain functional network with highly connected association cortical hubs. *J Neurosci* **26**, 63-72 (2006).
9. Humphries, M.D., Gurney, K. & Prescott, T.J. The brainstem reticular formation is a small-world, not scale-free, network. *Proc Biol Sci* **273**, 503-511 (2006).
10. Hagmann, P.*, et al.* Mapping the structural core of human cerebral cortex. *PLoS Biol* **6**, e159 (2008).
11. Tian, L., Wang, J., Yan, C. & He, Y. Hemisphere- and gender-related differences in small-world brain networks: a resting-state functional MRI study. *Neuroimage* **54**, 191-202 (2011).
12. Freeman, L.C. A set of measures of centrality based upon betweenness. *Sociometry* **40**, 35-41 (1977).
